# Molecular characteristics of screen-detected *vs* symptomatic breast cancers and their impact on survival

**DOI:** 10.1038/sj.bjc.6605317

**Published:** 2009-09-22

**Authors:** S J Dawson, S W Duffy, F M Blows, K E Driver, E Provenzano, J LeQuesne, D C Greenberg, P Pharoah, C Caldas, G C Wishart

**Affiliations:** 1 Department of Oncology, Strangeways Research Laboratories, University of Cambridge, Cambridge CB1 8RN, UK; 2 Cancer Research UK, Cambridge Research Institute, Li Ka Shing Centre, Robinson Way, Cambridge CB2 0RE, UK; 3 Cambridge Breast Unit, Addenbrooke's Hospital, Cambridge University Hospital NHS Foundation Trust, Cambridge CB2 2QQ, UK; 4 Cambridge Biomedical Research Centre, National Institute of Health Research, Cambridge CB2 2QQ, UK; 5 Cancer Research UK Centre for Epidemiology, Mathematics and Statistics, Wolfson Institute of Preventive Medicine, Barts and the London School of Medicine and Dentistry, Charterhouse Square, London EC1 M 6BQ, UK; 6 Eastern Cancer Registration and Information Centre, Unit C, Magog Court, Shelford Bottom, Hinton Way, Cambridge CB22 3AD, UK

**Keywords:** breast cancer, breast screening, survival, immunohistochemistry, biomarkers

## Abstract

**Background:**

Several recent studies have shown that screen detection remains an independent prognostic factor after adjusting for disease stage at presentation. This study compares the molecular characteristics of screen-detected with symptomatic breast cancers to identify if differences in tumour biology may explain some of the survival benefit conferred by screen detection.

**Methods:**

A total of 1379 women (aged 50–70 years) with invasive breast cancer from a large population-based case–control study were included in the analysis. Individual patient data included tumour size, grade, lymph node status, adjuvant therapy, mammographic screening status and mortality. Immunohistochemistry was performed on tumour samples using 11 primary antibodies to define five molecular subtypes. The effect of screen detection compared with symptomatic diagnosis on survival was estimated after adjustment for grade, nodal status, Nottingham Prognostic Index (NPI) and the molecular markers.

**Results:**

Fifty-six per cent of the survival benefit associated with screen-detected breast cancer was accounted for by a shift in the NPI, a further 3–10% was explained by the biological variables and more than 30% of the effect remained unexplained.

**Conclusion:**

Currently known biomarkers remain limited in their ability to explain the heterogeneity of breast cancer fully. A more complete understanding of the biological profile of breast tumours will be necessary to assess the true impact of tumour biology on the improvement in survival seen with screen detection.

Breast cancer is the most common female cancer in the UK with more than 45 000 women diagnosed per annum. The National Health Service Breast Screening Programme (NHSBSP) was introduced in 1988 in the UK and around 15 000 breast cancer cases are now detected every year through screening. The NHSBSP initially offered three yearly mammography to women aged 50–65 years, however, since 2004 the upper age limit has been increased to 70 years, with approximately two million women now screened in the UK each year.

The widespread introduction of mammographic screening for breast cancer has led to a significant reduction in breast cancer mortality. The policy of offering screening is associated with a 20% reduction in breast cancer mortality and the effect of actually attending screening is likely to be considerably larger (Vainio, 2002). Although women with screen-detected breast cancers have an improved prognosis compared with those diagnosed with tumours outside of screening (i.e. symptomatic tumours), there are multiple biases that may contribute to this difference. These include lead-time bias (cancers detected by screening are identified earlier in their natural history) and length bias (cancers with a favourable natural history are more likely to be detected by screening as they remain asymptomatic for longer; Shapiro *et al*, 1974; Zahl *et al*, 2004).

Screen-detected cancers are more likely to be smaller in size and well differentiated, and are less likely to be associated with regional lymph node involvement (Porter *et al*, 1999; Weaver *et al*, 2006). Until recently, the survival benefit conferred from screen detection has largely been attributed to this stage shift. However, several recent studies have shown that screen detection remains an independent prognostic factor after adjusting for disease stage (Joensuu *et al*, 2004; Shen *et al*, 2005; Wishart *et al*, 2008). In the largest UK series, which was performed by our group, only 72% of the survival benefit associated with screen-detected breast cancers was accounted for by age and shift in the Nottingham Prognostic Index (NPI), a prognostic tool based on tumour size, grade and lymph node status (Wishart *et al*, 2008). These results confirm that lead-time bias alone cannot fully explain the improved prognosis associated with screening. The remaining survival benefit conferred from screen-detection has not been fully explained, but may relate to differences in tumour biology between screen-detected and symptomatic cancers.

In recent years, microarray-based technology has resulted in the identification of clinically relevant molecular subtypes of breast cancer, providing insights into the molecular heterogeneity of the disease (Perou *et al*, 2000; Sorlie *et al*, 2003). Five distinct breast cancer subtypes have been identified based on gene expression signatures: luminal A, luminal B, HER2 overexpressing, basal-like and normal breast tissue-like. The differences in gene expression patterns among the subtypes reflect basic alterations in the cell biology of the tumours and have been associated with significant variation in clinical outcome. Luminal A tumours have been associated with the best prognosis whereas basal-like and HER2-overexpressing tumours show shorter disease-free intervals and worse overall survival (Sorlie *et al*, 2003).

Protein expression profiling using immunohistochemistry has been used to describe a similar molecular taxonomy of the disease (Callagy *et al*, 2003; Abd El-Rehim *et al*, 2005; Jacquemier *et al*, 2005). Luminal A and luminal B breast cancers express the oestrogen receptor (ER) and are also frequently progesterone receptor (PR) positive. HER2 expression is associated with the HER2-overexpressing and luminal B subtypes. Basal-like cancers are characteristically negative for ER, PR and HER2 expression (i.e. triple negative) but may express basal cytokeratins (CK5-6 and CK14) and the epidermal growth factor receptor (EGFR) (Nielsen *et al*, 2004; Rakha *et al*, 2006). In this context, immunohistochemistry can be used to identify distinct molecular subtypes of breast cancer, contributing to our understanding of the biological diversity of the disease.

It is currently not known whether variation in the molecular characteristics of screen-detected *vs* symptomatic breast cancers contributes to the differences seen in prognosis between these two groups. The aims of the present study were to examine the molecular characteristics of screen-detected *vs* symptomatic breast cancers and to identify if differences in tumour biology may explain some of the survival benefit conferred by screen detection.

## Materials and methods

### Study population

A total of 1379 women diagnosed with invasive breast cancer were included in this analysis from their participation in a large population-based case–control study; the Study of Epidemiology and Risk Factors in Cancer Heredity (SEARCH). Cases for SEARCH are identified through the Eastern Cancer Registration and Information Centre and details of the study have been published previously (Lesueur *et al*, 2005). The criteria for inclusion of cases in the current analysis were those aged between 50 and 70 years at diagnosis (the age range of the NHSBSP) with available tumour samples, pathology data (tumour size, tumour grade, lymph node status) and individual outcome data (vital status at last follow-up and date of death). The NPI was calculated for each case using tumour size, lymph node status and histological grade, and cases were classified into five different prognostic groups based on the NPI; excellent (NPI<2.4), good (2.4<NPI<3.4), moderate 1 (3.4<NPI<4.4), moderate 2 (4.4<NPI<5.4) and poor (NPI⩾5.4) (Sundquist *et al*, 1999).

### Tumour samples and immunostaining

Tissue microarrays were constructed using a tissue microarrayer (Beecher Instruments, Sun Prairie, WI, USA) as previously described with a single representative 0.6 mm tissue core taken from each tumour block (Kononen *et al*, 1998). Sections from the tissue microarrays were cut at 3 *μ*m for subsequent immunostaining. Immunostaining using the BOND-maX automated immunostainer (Leica Microsystems, Bucks, UK) was performed for the following 11 primary antibodies: ER, PR, HER2, CK 5-6, CK14, EGFR, E-cadherin, Ki-67, BCL2, p63 and *α*-smooth muscle actin (ASMA). Details of the primary antibodies and antigen retrieval methods are given in Supplementary Table 1. Tumours and tissues with known staining patterns were used as positive immunostaining controls and normal tissues were used a negative controls.

Following antibody staining, the slides were scanned into the Ariol high-throughput image analysis system (Applied Imaging Corp, San Diego, CA, USA). All immunohistochemistry was scored by SJD. Details of the scoring methods used and the cut-off values applied to each marker to indicate positive and negative staining are shown in Supplementary Table 1. Cores containing no evidence of tumour or carcinoma *in situ* were excluded from the analysis. Nuclear staining for ER and PR was recorded using the Allred scoring system; a simple additive scoring system of an intensity and proportion value that gives a range from 0 to 8 (intensity score range 0–3; proportion score range 0–5 (0=no staining; 1⩽1%; 2=1–10%; 3=11–33%; 4=34–66%; 5=67–100%)). A total score of >2 was defined as positive (Leake *et al*, 2000). HER2 was scored using the HercepTest; score 0=no staining or staining in ⩽10% cells, 1=weak incomplete membrane staining in >10% of cells, 2=moderate complete membrane staining in >10% cells, 3=strong complete uniform membranous staining in >10% of cells. A score of 2 or 3 was considered positive (Carlson *et al*, 2006). Scoring methods and cut-off values for the other immunohistochemical markers were defined from previous studies (Callagy *et al*, 2003, 2006; Rakha *et al*, 2006, 2007).

### Molecular subtype classification

Tumours were classified into five molecular groups based on the expression of ER, PR, HER2, basal cytokeratins and EGFR as follows: subtype 1 (luminal A)=ER+ and/or PR+ and HER2−; subtype 2 (luminal B)=ER+ and/or PR+ and HER2+; subtype 3 (HER2 over-expressing)=ER− and PR− and HER2+; subtype 4 (triple negative without expression of core basal markers)=ER−, PR−, HER2− and CK5/6−, CK14−, EGFR−; subtype 5 (triple negative with expression of core basal markers)=ER−, PR−, HER2− and CK5/6+ and/or CK14+ and/or EGFR+.

### Statistical analysis

Differences between screen-detected and symptomatic patients regarding categorical variables were assessed using the *χ*^2^-test. Cox regression analysis was used to determine the effect of each factor on all-cause mortality after diagnosis. Follow-up was censored on the date of death from any cause, or, if death did not occur, on the date last known alive or at 15 years after diagnosis, whichever came first. Time at risk began on the date of entry into SEARCH to allow for the fact that SEARCH is an ongoing epidemiology study and some cases are recruited after diagnosis (Azzato *et al*, 2009). The effect of screen detection as compared with symptomatic diagnosis on survival was first estimated unadjusted and then adjusted for NPI and individual marker expression. The percentage of the effect of screen detection accounted for by other factors was estimated using the measure mentioned by Freedman *et al* (1992). This is defined as 
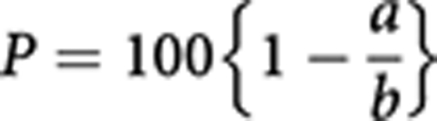
 where *b* is the unadjusted logarithm of the hazard ratio and *a* the adjusted. Thus, for example, if after adjustment there is no effect, *a*=0, and the Freedman statistic will be 100%. If the adjustment makes no difference whatever, *a*=*b*, and the Freedman statistic is 0%. All analyses were performed using SPSS statistical software version 16.0, or Intercooled Stata version 9.0 (StataCorp, College Station, TX, USA).

## Results

### Characteristics of breast cancers and mode of detection

Baseline clinical and pathology data from the 1379 women included in the analysis are summarised in Table 1. Of these women, 610 (44%) had screen-detected tumours and 769 (56%) had symptomatic tumours detected without screening. Mean follow-up of the study population was 7 years (range 0.7–16 years). Breast cancers identified through screening were smaller than those found without screening (85 *vs* 59% respectively had tumour size ⩽2 cm, *P*<0.0001). Screen-detected tumours were more likely to have lower histological grade (34 *vs* 23% were grade 1, *P*<0.0001) and less likely to have lymph node involvement (25 *vs* 40%, *P*<0.0001). In keeping with these findings, screen-detected tumours were more likely to have favourable NPI categories (*P*<0.0001). Women with screen-detected tumours were also less likely to receive adjuvant systemic chemotherapy (17 *vs* 30%, *P*<0.0001).

A higher proportion of tumours detected at screening were ER+ (86 *vs* 74%, *P*<0.0001)) and PR+ (74 *vs* 65%, *P*=0.002) (Table 2). BCL2 was highly expressed in screen-detected tumours (90 *vs* 83%, *P*=0.003) and lower rates of Ki-67 positivity (6 *vs* 15%, *P*<0.0001) were identified. There was no significant difference in the expression of the basal cytokeratins between the two groups but a higher proportion of tumours detected without screening expressed EGFR (6 *vs* 11%, *P*=0.012).

### Molecular subtypes of breast cancer and mode of detection

**Figure 1:**
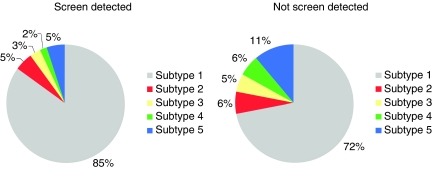
Breast cancer molecular subtypes by mode of breast cancer detection. Subtypes: 1=(ER+ and/or PR+) and HER2−; 2=(ER+ and/or PR+) and HER2+; 3=(ER− and PR−) and HER2+; 4=(ER−, PR−, HER2−) and (CK5/6−, CK14− and EGFR−); 5= (ER−, PR−, HER2−) and (CK5/6+ and/or CK14+ and/or EGFR+).

The distribution of the five defined molecular subtypes was explored in cancers detected at screening and those detected without screening (Table 2; Figure 1). Tumours detected at screening were more likely to belong to the luminal A subtype (subtype 1; 85 *vs* 72%, *P*<0.0001). Furthermore, screen-detected tumours were less likely to belong to the basal-like subtype as defined by either the triple negative phenotype (subtype 4; 2 *vs* 6%, *P*=0.005) or the triple negative phenotype and the expression of core basal markers (subtype 5; 5 *vs* 11%, *P*=0.001)). The prevalence of luminal B (7 *vs* 5%, *P*=0.62) and HER2-overexpressing subtypes (5 *vs* 3%, *P*=0.22) was similar between the screen-detected and non-screen-detected groups.

### Overall survival by mode of detection and molecular subtype

#### Univariate analysis

**Figure 2:**
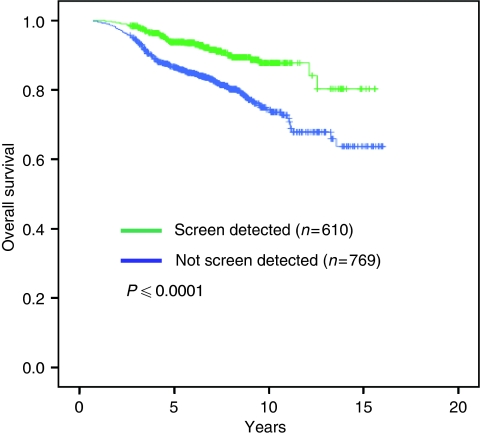
Overall survival by mode of breast cancer detection.

Women with screen-detected tumours had improved overall survival compared to women with tumours detected without screening (*P*<0.0001; Figure 2). Univariate analysis of mode of detection, age at diagnosis, tumour size, grade, nodal status and NPI group showed that all factors except age had highly significant effects on survival (Table 3).

When comparing survival according to molecular subtype, the luminal A subtype was associated with the best overall survival and the HER2-overexpressing subtype with the worst (Table 4). Tumours detected by screening generally had more favourable outcomes than non-screen-detected cancers regardless of the molecular subtype (Table 3). In the luminal A subtype (subtype 1), overall survival at 15 years was 94% among women with screen-detected cancers compared to 84% in the non-screen-detected group (*P*=0.001).

#### Multivariate analysis

The effect of screen detection on survival was then explored in a multivariate analysis after adjustment for tumour characteristics, the NPI, individual molecular biomarker expression and molecular subtype (Table 5). The effect of screen detection on survival was significantly attenuated after adjustment for the NPI with the hazard ratio changing from 0.43 to 0.69. Further adjustment of the model to incorporate expression of individual markers or the molecular subtype resulted in minimal change in the survival benefit conferred by screen detection.

The Freedman estimate of the proportion of the survival advantage from screen detection attributable to NPI was 56%. As one might argue that histological grade, a component of the NPI, is a biological variable, we also estimated the proportion attributable to size and node status only. This was 53%. Adjustment for the other molecular characteristics in addition to NPI gave attributable proportions ranging from 59 to 66%. Multivariate adjustment for several biological variables did not substantially change the proportion of the screen detection effect explained. Thus, additional adjustment for molecular characteristics explained a further 3–10% of the survival advantage of screen-detected tumours in addition to NPI (6–13% in addition to size and node status). Currently known biomarkers and tumour characteristics still remain limited in their ability to explain the heterogeneity of breast cancer fully. It is likely that a more complete understanding of the biological profile of breast tumours will be necessary to eliminate potential length bias and adjust for its impact on the improvement in survival seen with screen detection.

## Discussion

Women with screen-detected cancers have a strong survival advantage compared to women with symptomatic cancers detected without screening (Gotzsche and Nielsen, 2006). The majority of this survival benefit is explained by stage shift as tumours detected by screening are identified earlier in their natural history and consequently have an improved prognosis (Wishart *et al*, 2008). However, after adjustment for age and NPI, approximately one-third of the screen detection survival advantage has remained unexplained. It has been hypothesised that the remaining survival benefit conferred by screen detection may relate to differences in tumour biology between screen-detected and symptomatic cancers. We analysed the molecular characteristics of screen-detected *vs* symptomatic breast cancers in 1379 women diagnosed with invasive breast cancer in the East of England to identify if differences in tumour biology may explain some of the survival benefit conferred by screen detection. Although differences in the molecular subtype of screen-detected *vs* symptomatic cancers were identified, the expression of individual molecular biomarkers had minimal effect on the improved outcome associated with screen detection.

Favourable molecular characteristics have previously been described in screen-detected tumours including higher expression of ER and PR and less frequent expression of HER2 and Ki-67 (Crosier *et al*, 1999; Joensuu *et al*, 2004). Our current analysis confirmed these findings and identified that screen-detected tumours were more likely to be ER+, PR+ and Ki-67−. In our study, a low frequency of HER2+ breast cancer was identified, which is not an unexpected finding given that the cohort was population based. The proportion of HER2+ cases was found to be similar between the screen-detected and symptomatic tumours. Our analysis also identified that a higher proportion of breast cancers diagnosed without screening were triple negative. Symptomatic tumours were more likely to be EGFR+ although expression of the basal cytokeratins (CK5-6 and CK14) did not differ significantly between the screen-detected and symptomatic tumours. A previous small study of 95 interval cancers diagnosed in Norway has reported that cancers detected without screening were more likely to show a basal epithelial phenotype as defined by CK5-6 positivity (Collett *et al*, 2005). Finally, we identified that screen-detected tumours were more likely to show higher expression of BCL2, an anti-apoptotic protein whose expression is associated with improved survival from breast cancer (Callagy *et al*, 2006, 2008).

Despite the identification of distinct differences in the molecular characteristics of screen-detected *vs* symptomatic breast cancers, our analysis showed minimal attenuation of the screen-detected survival advantage after adjustment for the expression of individual molecular biomarkers or molecular subtype in multivariate analysis. The Freedman estimates of the proportion of the effect suggest that the NPI explains 56% of the effect, up to a further 10% is explained by the biological variables and more than 30% of the effect remains unexplained. In a recent study by Shito *et al* (2008), cancer detection at screening independently predicted favourable distant disease-free survival when molecular subtype was included as a covariate in multivariate analysis in addition to age, grade and tumour size. The authors concluded that the differences in molecular subtypes of screen-detected *vs* symptomatic breast cancers accounted in part for the better outcome of screen-detected cancers, however, the effect of molecular subtype on the survival advantage conferred by screen detection was not assessed in this analysis.

The policy of offering mammographic screening has led to a reduction in breast cancer mortality of around 20% (Vainio, 2002). The population mortality benefit occurs as a result of the much higher survival observed in screen-detected cancers. Around two-thirds of this survival benefit can be accounted for by age and shift in the NPI. The residual survival advantage from screen detection, although small, currently remains unexplained. Despite obvious differences in the molecular characteristics of screen-detected *vs* symptomatic tumours, our analysis indicates that these differences in tumour biology only account for a small proportion of the residual survival benefit. Measurement of tumour attributes is not perfect and there may be some residual variability in survival, which might be better explained by stage and other histological tumour characteristics if these were measured more precisely. In addition, there are prognostic factors that were unavailable to us, such as lymphovascular invasion. Adjustment for these might further explain some of the better survival of screen-detected cases. However, currently known biomarkers and tumour characteristics still remain limited in their ability to explain the heterogeneity of breast cancer fully. It is likely that a more complete understanding of the biological profile of breast tumours will be necessary to eliminate potential length bias and adjust for its impact on the improvement in survival seen with screen detection.

## Figures and Tables

**Table 1:** Case and tumour characteristics

	**Mode of breast cancer detection**		
**Characteristic**	**Screen detected (*n*=610)**	**Not screen detected (*n*=769)**	***P-*value**
*Age at diagnosis (years)*			
50–59	371 (61)	520 (68)	0.009
60–70	239 (39)	249 (32)	
Mean follow-up (years, range)	6.9 (1.3–15.6)	7.1 (0.7–16)	0.06
Deaths, *n* (%)	52 (9)	153 (20)	<0.0001
			
*Tumour size,* n *(%)*			
2 cm	482 (85)	429 (59)	<0.0001
>2–5 cm	84 (14)	284 (39)	
>5 cm	4 (1)	19 (2)	
			
*Grade,* n *(%)*			
1	181 (34)	153 (23)	<0.0001
2	277 (51)	296 (45)	
3	82 (15)	213 (32)	
			
*Nodal status,* n *(%)*			
Negative	400 (75)	426 (60)	<0.0001
Positive	134 (25)	290 (40)	
			
			
*NPI group,* n *(%)*			
Excellent	101 (25)	57 (10)	<0.0001
Good	132 (33)	112 (21)	
Moderate 1	100 (25)	123 (23)	
Moderate 2	46 (11)	147 (27)	
Poor	25 (6)	106 (19)	
			
*Morphology,* n *(%)*			
Ductal	454 (74)	547 (71)	0.05
Lobular	102 (17)	159 (21)	
Other	54 (9)	61 (8)	
			
			
*Chemotherapy,* n *(%)*			
Yes	83 (17)	204 (30)	<0.0001
No	406 (83)	474 (70)	
			
*Hormonal therapy,* n *(%)*			
Yes	543 (89)	656 (85)	0.10
No	67 (11)	112 (15)	

Abbreviation: NPI=Nottingham Prognostic Index.

**Table 2:** Molecular characteristics

	**Mode of breast cancer detection**		
**Characteristic**	**Screen detected (*n*=610)**	**Not screen detected (*n*=769)**	***P*-value**
*ER status,* n *(%)*			
Positive	352 (86)	410 (74)	<0.0001
Negative	56 (14)	144 (26)	
			
*PR status,* n *(%)*			
Positive	302 (74)	350 (65)	0.002
Negative	106 (26)	191 (35)	
			
*HER2 status,* n *(%)*			
Positive	34 (8)	63 (12)	0.10
Negative	371 (92)	478 (88)	
			
*CK5-6 status,* n *(%)*			
Positive	44 (11)	52 (10)	0.65
Negative	371 (89)	483 (90)	
			
*CK14 status,* n *(%)*			
Positive	16 (4)	20 (4)	0.83
Negative	392 (96)	528 (96)	
			
*EGFR status,* n *(%)*			
Positive	25 (6)	60 (11)	0.012
Negative	367 (94)	477 (89)	
			
*E-cadherin status,* n *(%)*			
Positive	303 (74)	415 (75)	0.65
Negative	107 (26)	137 (25)	
			
*Ki-67 status,* n *(%)*			
Positive	26 (6)	86 (15)	<0.0001
Negative	382 (94)	473 (85)	
			
*BCL2 status,* n *(%)*			
Positive	360 (90)	442 (83)	0.003
Negative	42 (10)	93 (17)	
			
*p63 status,* n *(%)*			
Positive	3 (1)	2 (1)	0.43
Negative	403 (99)	543 (99)	
			
*ASMA status,* n *(%)*			
Positive	18 (4)	26 (5)	0.75
Negative	390 (96)	510 (95)	
			
Subtype 1, *n* (%)^a^	322 (85)	366 (72)	<0.0001
Subtype 2, *n* (%)	21 (5)	32 (6)	0.62
Subtype 3, *n* (%)	12 (3)	24 (5)	0.216
Subtype 4, *n* (%)	7 (2)	28 (6)	0.005
Subtype 5, *n* (%)	19 (5)	56 (11)	0.001

Abbreviations: ASMA=*α*-smooth muscle actin; EGFR=epidermal growth factor receptor; ER=oestrogen receptor; NPI=Nottingham Prognostic Index; PR=progesterone receptor.

a Subtypes: 1=(ER+ and/or PR+) and HER2−; 2=(ER+ and/or PR+) and HER2+; 3=(ER− and PR−) and HER2+; 4=(ER−, PR−, HER2−) and (CK5/6−, CK14− and EGFR−); 5=(ER−, PR−, HER2−) and (CK5/6+ and/or CK14+ and/or EGFR+).

**Table 3:** Univariate analysis of overall survival

	**Hazard ratio (95% CI)**	***P*-value**
*Mode of detection (*n=*1379)*		
Not screen detected	1.00	
Screen detected	0.43 (0.31–0.59)	<0.0001
		
*Age at diagnosis (*n=*1400)*		
50–59	1.00	
60–70	1.01 (0.74–1.35)	0.99
		
*Tumour size (*n=*1319)*		
2 cm	1.00	
>2–5 cm	2.11 (1.56–2.85)	<0.0001
>5 cm	5.88 (3.23–10.73)	<0.0001
		
*Grade (*n=*1218)*		
1	1.00	
2	1.98 (1.21–3.23)	0.006
3	5.42 (3.35–8.75)	<0.0001
		
*Nodal status (*n=*1267)*		
Negative	1.00	
Positive (1–3 nodes)	2.35 (1.65–3.35)	<0.0001
Positive (4 nodes)	8.18 (5.75–11.65)	<0.0001
		
*NPI group (*n=*961)*		
Excellent	1.00	
Good	3.22 (1.10–9.47)	0.034
Moderate 1	4.70 (1.64–13.52)	0.004
Moderate 2	7.36 (2.61–20.77)	<0.0001
Poor	27.77 (10.09–76.41)	<0.0001

Abbreviation: NPI=Nottingham Prognostic Index.

**Table 4:** Overall survival by breast cancer molecular subtype

		**Overall survival (15 years)**		
**Molecular subtype**	***n***	**Screen detected (%)**	**Not screen detected (%)**	***P*-value**
Subtype 1^a^	688	94	84	0.001
Subtype 2	53	86	78	0.52
Subtype 3	36	83	54	0.12
Subtype 4	35	86	64	0.24
Subtype 5	75	84	79	0.60

a Subtypes: 1=(ER+ and/or PR+) and HER2−; 2=(ER+ and/or PR+) and HER2+; 3=(ER− and PR−) and HER2+; 4=(ER−, PR−, HER2−) and (CK5/6−, CK14− and EGFR−); 5=(ER−, PR−, HER2−) and (CK5/6+ and/or CK14+ and/or EGFR+).

**Table 5:** Attenuation of the effect of screen detection on survival after adjustment for different factors

**Factors adjusted for**	**Hazard Ratio (95% CI)** **Screen detected *vs* not screen detected**	***P*-value**	**Freedman statistic (%)**
None	0.43 (0.31–0.59)	<0.0001	—
Grade	0.50 (0.35–0.71)	<0.0001	18
Size	0.52 (0.37–0.74)	<0.0001	22
Nodal status	0.60 (0.43–0.84)	0.003	39
Size and nodal status	0.67 (0.47–0.94)	0.02	53
NPI	0.69 (0.46–1.03)	0.07	56
NPI and ER	0.71 (0.45–1.14)	0.16	59
NPI and PR	0.72 (0.45–1.16)	0.18	61
NPI and HER2	0.72 (0.45–1.15)	0.16	61
NPI and CK5-6	0.75 (0.47–1.19)	0.22	66
NPI and CK14	0.68 (0.43–1.09)	0.11	54
NPI and EGFR	0.63 (0.38–1.03)	0.07	45
NPI and E-cadherin	0.65 (0.41–1.05)	0.08	49
NPI and Ki-67	0.72 (0.45–1.14)	0.08	61
NPI and BCL2	0.71 (0.44–1.15)	0.16	59
NPI and p63	0.59 (0.36–0.94)	0.03	37
NPI and ASMA	0.72 (0.45–1.14)	0.16	61
NPI and molecular subtype	0.64 (0.38–1.08)	0.09	47

Abbreviations: ASMA=*α*-smooth muscle actin; EGFR=epidermal growth factor receptor; ER=oestrogen receptor; NPI=Nottingham Prognostic Index; PR=progesterone receptor.
