# Supplementary material for: Molecular characteristics of screen-detected vs symptomatic breast cancers and their impact on survival
Source: Br J Cancer. 2009 Sep 22;101(8):1338–44. doi: 10.1038/sj.bjc.6605317 (PMC2768460; doi:10.1038/sj.bjc.6605317)
Supplement: Supplementary Table 1 [file 6605317x1.doc]

**Table 1: Primary Antibodies**

| **Antibody** | **Source** | **Dilution** | **Antigen retrieval^*^** | **Scoring** | **Cutoff for positivity** |
| --- | --- | --- | --- | --- | --- |
| **ER**  **PR**  **HER2**  **CK 5-6**  **CK 14**  **EGFR**  **E-Cadherin**  **Ki-67**  **BCL2**  **p63**  **ASMA** | Novocastra  Clone 6F11/2  Dako  Clone PgR 636  Dako c-erbB-2  Dako  Clone D5/16 B4  Novocastra  Clone LL002  Zymed  Clone 31G7  Dako  Clone NCH-38  Dako  Clone MIB-1  Dako  Clone 124  Novocastra  Clone 7JUL  Sigma  Clone IA4 | 1:70  1:50  1:250  1:50  1:20  1:25  1:25  1:200  1:200  1:50  1:2000 | Citrate buffer  pH 6, 30 minutes  Citrate buffer  pH 6, 30 minutes  Citrate buffer  pH 6, 40 minutes  Tris-EDTA buffer  pH 9, 30 minutes  Citrate buffer  pH 6, 30 minutes  Proteinase K  1 unit, 10 minutes  Citrate buffer  pH 6, 30 minutes  Tris-EDTA buffer  pH 9, 30 minutes  Tris-EDTA buffer  pH 9, 20 minutes  Tris-EDTA buffer  pH 9, 30 minutes  No antigen retrieval | Allred score^**^  Allred score  Herceptest^***^  0=negative  1=positive  0=negative  1=positive  Allred score  0=negative  1=positive  Proportion score as for Allred system: 0-5  Intensity score; 0=negative, 1=weak, 2=moderate, 3=strong  Proportion score; 0-100%  0=negative  1=positive  0=negative  1=positive | Total score >2  Total score >2  Scores 2 and 3  ≥10% of cells showing positive staining  ≥10% of cells showing positive staining  Total score >2  ≥10% membranous staining regarded as positive  >10% of cells showing positive staining (i.e. score ≥ 3)  Staining intensity of ≥1 and ≥10% cells staining positively  ≥10% of cells showing positive staining  ≥10% of cells showing positive staining |

^*^All antigen retrieval was performed using the BOND^TM^ Polymer Refine detection kit

^**^ Allred Score: an additive scoring system of an intensity and proportion value that gives a range from 0 to 8 (intensity score range 0-3; proportion score range 0-5 (0=no staining; 1=<1%; 2=1%-10%; 3=11%-33%; 4=34%-66%; 5=67%-100%)).

^***^ Herceptest: score 0=no staining or staining in ≤10% cells, 1=weak incomplete membrane staining in >10% of cells, 2=moderate complete membrane staining in >10% cells, 3=strong complete uniform membranous staining in >10% of cells.
